# Synthesis, Linear and Nonlinear Optical Properties of Ag/Al_2_O_3_ Nanocomposites

**DOI:** 10.3390/ma15186322

**Published:** 2022-09-12

**Authors:** Sharafudeen Kaniyarakkal, Tiny Thomas, Saravana Kumar Sadagopalan, Lekshmi Jayamohan, Remya Muralimanohar, Lekshmi Vasanthakumaryamma, Vijayakumar Sadasivan Nair

**Affiliations:** 1 Department of Physics, Kuwait College of Science and Technology, Doha 35004, Al-Asimah, Kuwait; 2 PG and Research Department of Physics, NSS College Pandalam, Pathanamthitta 689501, Kerala, India; 3 PG and Research Department of Physics, Christian College Chenganoor, Alappuzha 689122, Kerala, India; 4 NSS Training College Ottapalam, Palakkad 679101, Kerala, India

**Keywords:** nanocomposites, nonlinear absorption, surface plasmon resonance, two-photon absorption

## Abstract

This work reports a detailed study of the synthesis, characterization and third-order nonlinear optical properties of Ag and Al_2_O_3_ nanoparticles and their polymer nanocomposites. Ag and Al_2_O_3_ nanoparticles were prepared by the chemical precipitation method. The X-ray diffraction studies confirmed the purity and the crystalline nature of the sample and revealed the crystallite size. The linear optical properties and the structural morphology of the nanoparticles were confirmed using UV–visible spectroscopy and SEM analysis. The prepared nanoparticles were introduced into the polymer matrix by the spin-coating technique. Open-aperture and closed-aperture Z-scan technique was used to study the nonlinear absorption and nonlinear refraction of the samples under a Q-switched Nd:YAG laser at 532 nm. The observed third-order nonlinear optical susceptibility (χ^(3)^) was on the order of 10^−6^ esu, which indicates that these materials are potential candidates for photonic applications.

## 1. Introduction

Research in the field of nonlinear optical (NLO) materials has led to the development of optical devices with applications such as ultrafast optical switching, optical limiting, optical communications and high-density optical storage [1,2,3]. From a wide range of nonlinear optical materials, nanomaterials that encompass metals, semiconductors, carbon nanotubes and carbon nanowires have recently attracted significant attention due to the excellent tunability of their nonlinear optical properties [4,5,6]. Nanomaterials with nonlinear optical responses can also be used as contrast agents in the field of nonlinear microscopy and optical limiting applications [7]. The optical interaction via Surface Plasmon Resonance (SPR) enhances the nonlinear optical response of metal nanoparticles associated with random media. Therefore, metal nanoparticles embedded in various host systems are widely chosen for optoelectronic applications. Specifically, silver (Ag) and alumina (Al_2_O_3_) show large third-order nonlinear optical properties that make them suitable for various nonlinear optical applications [8,9]. In the case of Ag nanoparticles, there is a 3.9 eV separation between the interband transition edge and plasmon resonance, which brings a strong influence in the dispersion of effective third-order nonlinearity around localized surface plasmon resonance. On the other hand, in Au and Cu nanoparticles, the dependence on the dispersion of effective third-order nonlinearity is not seen due to the overlapping of these levels. This paves the way for tuning the internal local electric field in Ag nanoparticle-based systems using nonlinearity to favour specific nonlinear optical applications in them by controlling and optimizing the nanoparticle’s size and shape [10,11,12]. Jacob et al. demonstrated that the shape and size of metallic inclusions can be optimized in nonlinear optical materials for enhanced performance, and such materials that rely on thermally induced nonlinearity can find use in ultrafast optical devices that can operate in low power [13]. Del Coso et al. studied the nonlinear optical studies of Cu:Al_2_O_3_ nanocomposite film using degenerate four-wave mixing and found it to have enhanced optical nonlinearity as high as 1.8 × 10^−7^ esu; underlying mechanisms were attributed to high local resonance effects and particles’ multipolar interactions, which originated from the particle’s coalescence and the size effect of the nanocrystals [14]. The studies on samples based on multiple Al_2_O_3_/ZnO bilayers in silica demonstrated a large positive nonlinear refractive index, which can be controlled via fabrication parameters used in the atomic layer deposition (ALD) technique [15]. Bakkali et al. investigated discontinuous multilayer (DML) films with a composition of a homogeneous layer of Au−Al_2_O_3_ nano-granular metals and found interesting optical characteristics due to the electromagnetic interactions that originated from the localized surface plasmon resonance, which referred to the size, shape and surrounding media of the Au nanoparticles [16]. There is a dearth of data about the third-order nonlinear optical studies of Ag/Al_2_O_3_ composites in femtosecond, picosecond and nanosecond laser pulse regimes [17]. In this work, Polyvinyl alcohol (PVA) was selected as a host for Ag/Al_2_O_3_ nanocomposites because it is environmentally friendly and possesses distinguishing properties such as water solubility and good mechanical strength [18,19,20]. Nonlinear optical studies on CdSe quantum dots-based Al_2_O_3_/Ag composite films were done and showed the significant nonlinear optical effects in them under femtosecond laser excitation [21]. So far, the intense laser–matter interaction in the Ag/Al_2_O_3_/polymer composite system has not yet been studied.

This work presents a detailed study of the synthesis, characterization and third-order nonlinear optical properties of the selected metal nanoparticles and the Ag/Al_2_O_3_/PVA polymer nanocomposite. The structural, linear and nonlinear optical characterizations of the newly synthesized nanomaterials are explored using various characterization techniques. A Q-switched Nd:YAG laser at 532 nm was used as the source of light in the Z-scan method and was used to extract and compare the third-order nonlinear optical susceptibility (χ^(3)^) of the samples [22].

## 2. Materials and Methods

### 2.1. Synthesis

Silver and Al_2_O_3_ nanoparticles were prepared by a simple chemical precipitation method [23]. An amount of 0.01 N aqueous silver nitrate solution was added to 100 mL of distilled water and heated up to 80 °C. Lemon juice extract was taken and filtered using Whatman filter paper, and the filtered lemon juice was added dropwise to the silver nitrate solution. Subsequently, the colour of the solution changed from clear to pale yellow to an orange colour, indicating the presence of silver nanoparticles. The obtained precipitate was then dried and powdered. Aluminium chloride was used as the precursor for the preparation of Al_2_O_3_ nanoparticles. An amount of 1 M aqueous solution of Al_2_Cl_2_ was mixed with distilled water and stirred for 5 min. A known amount of Triethanolamine (TEA) was added to the solution as a capping agent and stirred for minutes. Then, 1 M 15 mL as-prepared NaOH solution was added to the above solution and stirred for 30 min in a magnetic stirrer to obtain a homogeneous solution. After 30 min, a white colour precipitate was obtained and was separated by centrifugation at 3000 rpm for 20 min up to 6 times with distilled water and finally with acetone. The precipitate was separated by centrifugation with distilled water and acetone. The precipitate was dried and powdered and then annealed at 850 °C for 6 h in a Muffle furnace.

To prepare Ag/PVA thin films, 2 wt% of Ag was dissolved in 20 mL of PVA solution, and the final solution was stirred using a magnetic stirrer for 30–40 min to prepare a homogeneous mixture. This solution was sonicated in an ultrasonicator for 5 min to ensure the dispersion of the particles. Then, the solution was made into thin films by spin-coating in a well-cleaned flat glass substrate at 1000 rpm for 1 min [24]. To prepare Al_2_O_3_/PVA films, 2 wt% of Al_2_O_3_ was dissolved in 20 mL of PVA solution, and the same procedures were repeated [25,26]. For Ag/Al_2_O_3_/PVA films, both Ag and Al_2_O_3_ were dissolved in 20 mL of PVA in 1:4 and 4:1 ratios, and the same procedures were repeated to form thin films.

### 2.2. Characterization

UV–visible spectroscopy (Systronics, Ahmedabad, India) was employed to study the linear optical properties, such as the absorption peak and band gap, of the nanoparticles. The morphology and structure were analysed by SEM. The X-ray diffraction studies confirmed the purity and the crystalline nature of the sample and also revealed the crystallite size.

The Z-scan technique was used to determine the nonlinear optical properties of the samples. The source used was an Nd-YAG laser with the output beam having Gaussian spatial and temporal profiles operating at a wavelength of 532 nm. Open-aperture and closed-aperture Z-scan experiments were done to measure the nonlinear absorption coefficient (β) and nonlinear refractive index (γ). The absolute value of third-order nonlinear optical susceptibility (χ^(3)^) was extracted from the real and imaginary parts of susceptibility.

## 3. Results and Discussion

**Figure 1:**
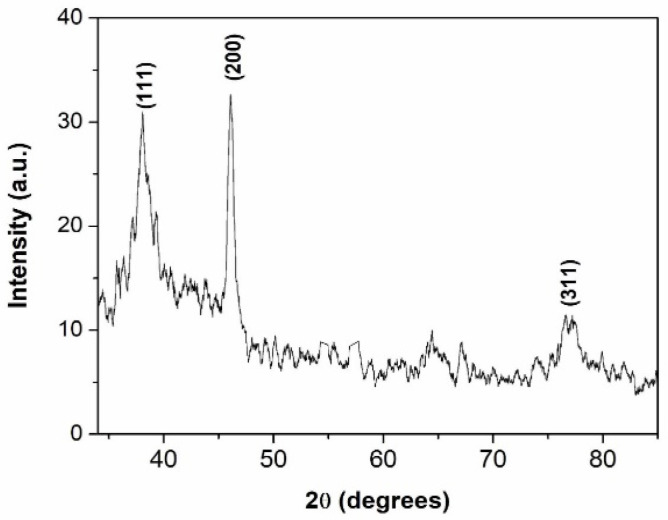
XRD pattern of nanoparticles of Silver.

**Figure 2:**
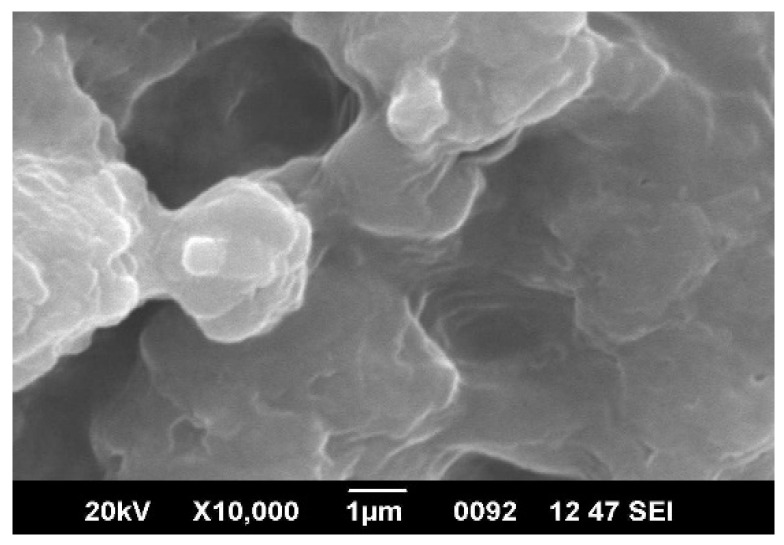
SEM images of nanoparticles of Silver.

Figure 1 shows the XRD pattern of the prepared Ag nanoparticles. The figure shows three diffraction peaks at 2θ values of 38.07°, 46.02° and 77.15° corresponding to d-values of 2.362 Å, 1.971 Å and 1.24 Å, respectively. The peaks were identified to originate from the (111), (002) and (311) planes of the cubic phase of silver. The grain size of Ag nanoparticles calculated using the Debye–Scherrer equation was approximately 40 nm. Figure 2 shows the SEM images of Ag nanoparticles, from which it can be observed that the silver nanoparticles were spherical and agglomerated.

**Figure 3:**
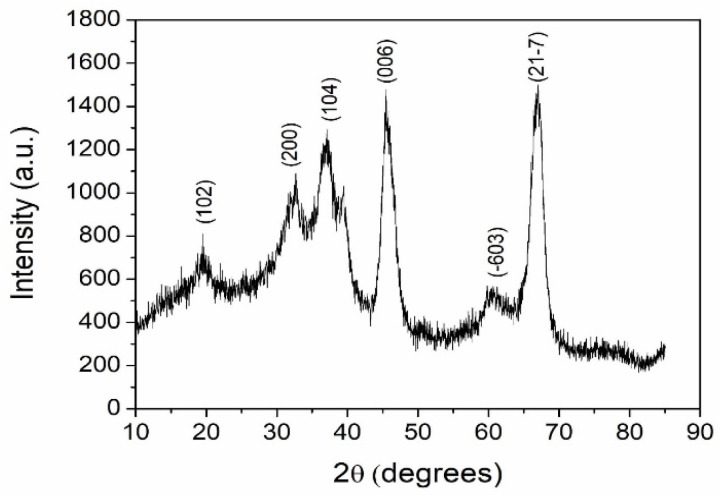
XRD pattern of Al_2_O_3_ nanoparticles.

**Figure 4:**
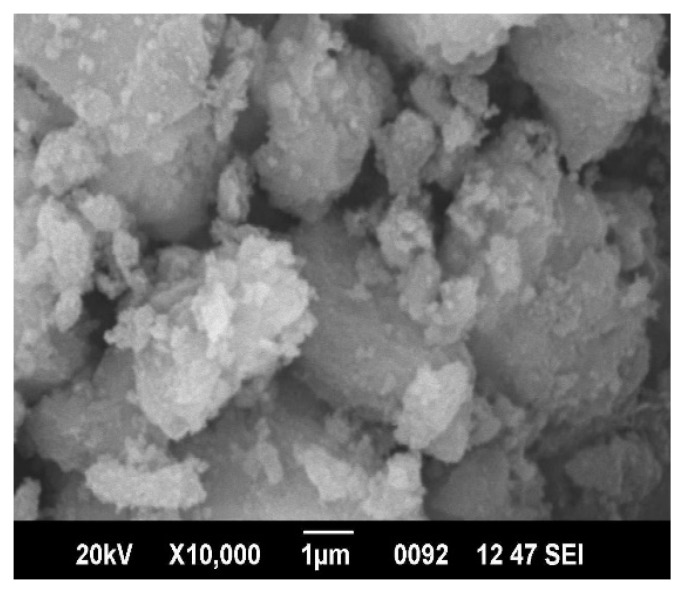
SEM images of Al_2_O_3_ nanoparticles.

The XRD pattern of Al_2_O_3_ nanoparticles is shown in Figure 3. The d-values corresponding to each peak in the XRD pattern were calculated and compared with the standard JCPDS values for Al_2_O_3_. The 2θ values of diffraction peaks in the present study matched with the monoclinic structure and θ phase of aluminium oxide. The grain size of the nanoparticles of aluminium oxide calculated from the Debye–Scherrer equation was approximately 5 nm. The SEM images of nanoparticles of Al_2_O_3_ are shown in the Figure 4. The SEM images reveal that the nanoparticles were slightly agglomerated and had an irregular shape.

**Figure 5:**
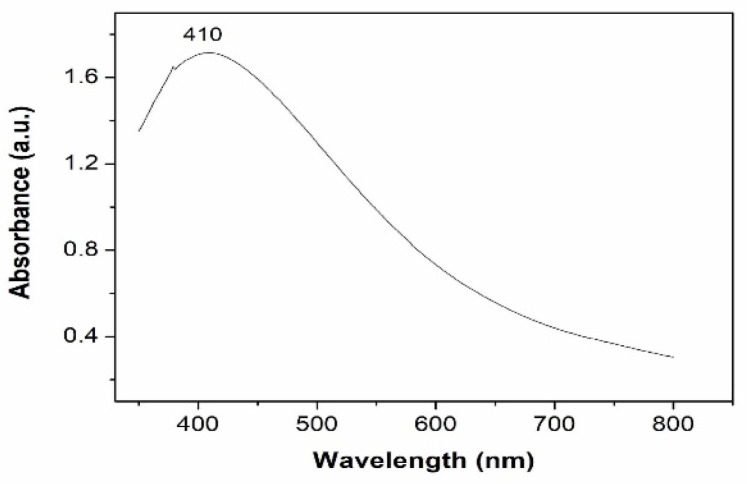
UV–visible absorption spectrum of as-prepared Ag nanoparticles.

**Figure 6:**
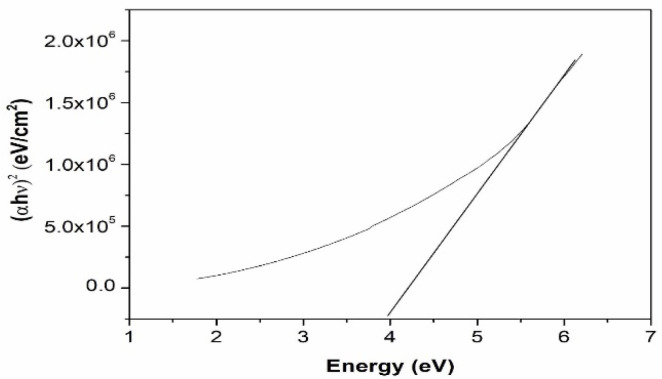
Plot of (αhν)^2^ vs. hν of Al_2_O_3_ nanoparticles.

**Figure 7:**
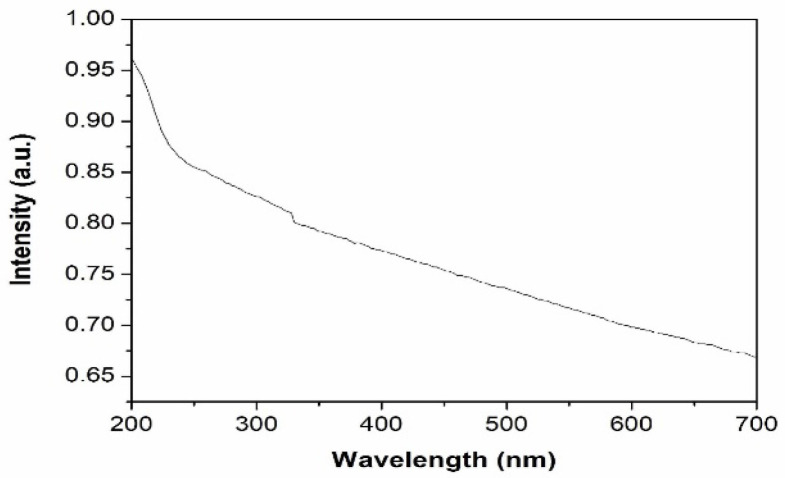
Absorption spectrum of Al_2_O_3_ nanoparticles.

The UV–visible absorption spectrum of the synthesized nanoparticles is shown in Figure 5. The spectrum exhibits a broad peak around 410 nm. Noble metal nanoparticles are known to exhibit unique optical properties due to the property of Surface Plasmon Resonance (SPR), which is the collective oscillation of the free electrons when subjected to an external field and may vary depending on the shape, size and surrounding of the particle. The peak observed in the absorption spectrum of silver nanoparticles in the present study can be ascribed to absorption due to surface plasmon resonance. The UV–visible absorption spectrum and the plot of (αhν)^2^ vs. hν of Al_2_O_3_ nanoparticles in the system are shown in Figure 6 and Figure 7, respectively. The band gap of aluminium oxide nanoparticles was found to be 4 eV, which is low compared to that of the bulk band gap (6.6 eV). The decrease in band gap can be attributed to the localized electronic levels in the band gap of the material.

The nonlinear optical properties of the samples were studied using the Z-scan technique. The input energy used was 100 mW, operating at a wavelength of 532 nm. The source used was an Nd-YAG laser with the output beam having Gaussian spatial and temporal profiles. Open- and closed-aperture Z-scan experiments were performed with an aperture placed in front of the detector at a distance far from the Rayleigh diffraction length (Z_0_) of the focused laser beam.

**Figure 8:**
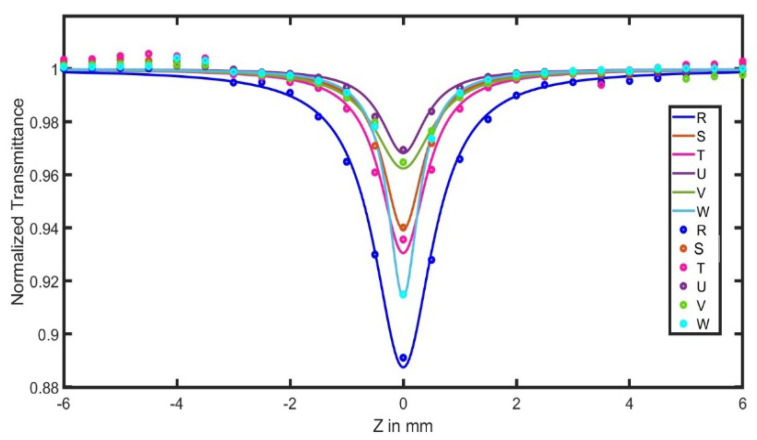
Open-aperture Z-scan curves for the samples: R (Ag), W (Al_2_O_3_), V (Al_2_O_3_/PVA film), S (Ag/PVA film), T (Ag/Al_2_O_3_/PVA1 film), U (Ag/Al_2_O_3_/PVA2 film).

Figure 8 shows the normalized transmission for the open-aperture (OA) Z-scan. Here, the solid line is a theoretical fit of the experimental data according to the equation:

T(z) =∑m=0∞[−q0(z)]m(m+1)32

These fitted data yield the effective two-photon absorption coefficient (TPA) value, β. The transmittance showed a minimum value at the focus Z = 0 and then increased steadily on both sides of the focus, which is represented by a valley indicating the reverse saturable absorption (RSA). The RSA and saturable absorption (SA) effects are two competing nonlinear responses that require the high-intensity laser and are dependent on laser wavelength, pulse width, laser polarization and the intrinsic properties of specific materials [27]. From the data, a symmetric valley represents a positive nonlinear absorption coefficient β for all types of nanocomposites, which indicates the occurrence of two-photon absorption.

**Figure 9:**
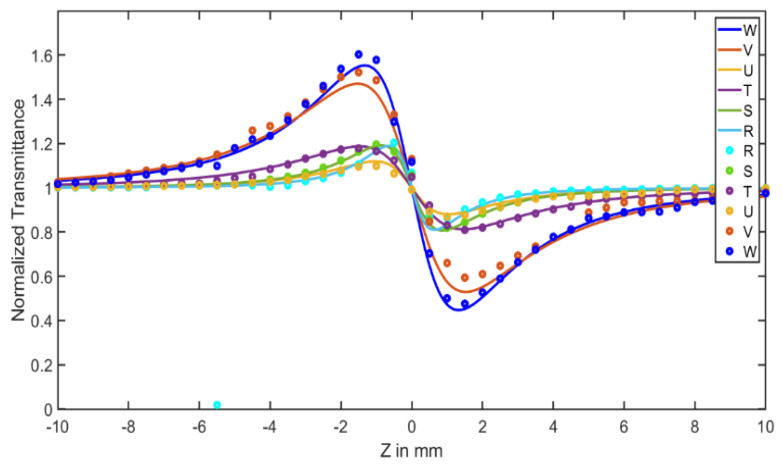
Pure nonlinear curves for the samples: R (Ag), W (Al_2_O_3_), V (Al_2_O_3_/PVA film), S (Ag/PVA film), T (Ag/Al_2_O_3_/PVA1 film), U (Ag/Al_2_O_3_/PVA2 film).

In the pure nonlinear refraction curve shown in the Figure 9, the experimental data are fitted according to the equation:

$$T\left(z\right)=1-\frac{4\Delta \phi_{0}x}{\left(1+x^{2}\right)\left(9+x^{2}\right)}$$

where x is the normalized distance, which is related to the sample movement distance (z) through x=zz0 with $z_{0}$ being the Rayleigh length; $\Delta \phi_{0}$ is the nonlinear phase shift, which is related to the nonlinear refractive index through $n_{2}=\frac{\Delta \phi_{0}}{{\mathrm{kL}}_{\mathrm{eff}}I_{0}}$, where k=2πλ is the wave vector; $\left|\Delta \phi_{0}\right|$ can be found from the fitted open curve $\Delta T_{P-V}=0.406{\left(1-S\right)}^{0.25}\left|\Delta \varphi_{0}\right|$, in which $\Delta T_{P-V}=T_{P}-T_{V}$ represents the difference in the normalized transmittance of the peak–valley in the normalized Z-scan curve.

The peak–valley configuration of the pure nonlinear curve obtained by the division of the samples revealed a self-defocusing effect, and the sign of the nonlinear refractive index was negative. The sample was considered as a thin negative lens because the pre-focus maximum was followed by a post-focus minimum.

The calculated values of the third-order optical parameters for the samples are given in the Table 1. The result shows that the samples exhibited third-order nonlinear optical properties. The real part (Reχ^(3)^) and imaginary part (Imχ^(3)^) of the susceptibility were on the order of 10^−6^ esu. The calculated value of third-order nonlinear susceptibility χ3=(Reχ(3))2+(Imχ(3))2 and nonlinear refractive index (n_2_) for the samples were on the order of 10^−6^ esu and 10^−9^ esu, respectively. The observed nonlinear refractive index values were larger compared to the value reported for Al_2_O_3_ bulk in the literature, which is around γ = 1.33 × 10^−9^ cm^2^/W, and Al_2_O_3_/ZnO multilayer films, which is around γ = 2.50 × 10^−9^ cm^2^/W [15,28]. These values of third-order nonlinear susceptibility in the samples are comparable to the value reported for the metal Au/ZnO nanoparticle arrays and CuO/ZnO/PVA nanocomposites in the literature [18,29]. The third-order nonlinear optical response in their samples was shown to be due to the surface plasmon oscillation of the metal nanoparticles. In another work, it was noted that Ag/PVA nanocomposites exhibited third-order nonlinear susceptibility values on the order of 10^−7^ to 10^−6^ esu [30]. In the present work, the third-order nonlinear optical response of the nanocomposite films was investigated with various compositions. The Al_2_O_3_/PVA film exhibited a third-order nonlinearity of 2.49 × 10^−6^ esu, which is comparable to the values reported in the literature [25]. Slightly higher values were seen in the sample when PVA was used as the polymer instead of PVC. When the metal nanoparticles induce the local field due to surface plasmon interaction, the nonlinear enhancement effect is strengthened, thus increasing the nonlinear behaviour of the composite film. The oxygen vacancies in Al_2_O_3_ are most probably defects that introduce gap levels inducing further energy transfers. Moreover, as a polarization medium, Al_2_O_3_ interacts with Ag NPs to enhance the polarization field of the composite system, which significantly improves the nonlinear optical parameters of the composite films. The nonlinear parameters of the nanocomposite films with Ag/Al_2_O_3_ incorporated in PVA were tabulated, and it proved the tunability of the third-order nonlinear response with varying concentrations.

The sign of n_2_ for the prepared nanocomposites was negative, indicating that the samples exhibited a self-defocusing effect, and hence, it diverged the radiation passing through it. This can happen due to the thermal lens effect, the electrostriction effect or the optical Kerr effect under the action of an increased timescale of nanosecond laser pulses when compared to short, femtosecond timescale pulses. In all conventional nonlinear optical materials, the normalized nonlinear index change (Δn/n) under an ultra-fast regime is less than 1% due to a saturation of nonlinearity, as higher-order susceptibility contributions leap in and optical breakdown occurs [31,32]. This small nonlinear index change is due to fast nonlinearity originating from slight changes in electronic clouds, termed “electronic” contribution. However, the electron cloud’s confined binding potential is unaffected due to the unaffected crystal field or crystal bond. In contrast to that, when the temperature raises, the binding potential changes due to the increase in the atom–atom distances, which leads to an increase in the nonlinear refractive index and, hence, a higher thermo-optical coefficient. This can result in the fast motion of ions, typically a few hundred femtoseconds, in accordance with the optical phonon frequency. However, in practice, the thermal nonlinearity is so slow, on the order of a few microseconds, due to heat dissipation. However, this can be untrue if the heated region is relatively small, and hence, metal nanoparticles such as Ag with SPPs can confine the optical filed in a nanometre region; hence, the temperature dissipation is minimized, which can lead to a large, thermally induced nonlinear refractive index change with responses on the order of a few picoseconds or nanoseconds. Thus, the localization of the optical field due to nanometal impregnation can bring faster timescale diffusive processes, such as thermal diffusion or carrier diffusion [33,34]. In the present study, the thermal contribution of nonlinearity is minimal since the Ag nanoparticle’s confined optical field is not significantly high because of the non-resonant excitation laser wavelength, as well as the low concentration of the nanometals in the system. Moreover, the operating pulse peak power was low to avoid damage in the sample due to optical breakdown, and the employed excitation laser was not in CW operation. It can also be noted that the presence of a strong nonlinear absorption related to the imaginary part of the third-order susceptibility also showed good optical limiting. These results reveal that these samples are suitable candidates for various photoelectronic applications.

## 4. Conclusions

Ag and Al_2_O_3_ nanoparticles were synthesized by the co-precipitation method, and their composite films were prepared by the spin-coating method. Their optical and structural properties were revealed using UV–visible absorption, XRD and SEM. The open-aperture Z-scan studies revealed a positive nonlinear absorption for all types of samples that originated from two-photon absorption. The shape of the pure nonlinear curve confirmed the self-defocusing effect. The samples exhibited impressive third-order nonlinear susceptibility (χ^(3)^) on the order of 10^−6^ esu. The results suggest that this material could find use in nonlinear optical devices.

## Figures and Tables

**Table 1:** Nonlinear optical parameters of different samples.

Sample	Sample Symbol	γ × 10^−9^ (cm^2^/W)	β × 10^−4^ (cm/W)	Re χ^(3)^ × 10^−6^ (cm^2^/W)	Im χ^(3)^ × 10^−6^ (cm/W)	χ^(3)^ × 10^−6^ (esu)
Ag	R	5.26	3.51	5.55	1.11	5.66
Al_2_O_3_	W	5.57	3.79	5.78	1.31	5.93
Ag/PVA	S	2.25	2.80	2.33	1.68	2.87
Al_2_O_3_/PVA	V	2.10	2.67	2.02	1.46	2.49
Ag/Al_2_O_3_/PVA1	T	2.40	3.07	2.85	1.32	3.14
Ag/Al_2_O_3_/PVA2	U	2.92	3.16	3.03	1.65	3.46

## Data Availability

Data underlying the results presented in this paper are not publicly available at this time but may be obtained from the corresponding author upon reasonable request.
